# Evaluation of the Use of Artificial Intelligence in Dental Imaging Interpretation: A Systematic Review

**DOI:** 10.7759/cureus.109628

**Published:** 2026-05-25

**Authors:** Madura C Nadarge, Deepa A Das

**Affiliations:** 1 Oral Medicine and Radiology, Dr. Gajanan Dattatray (GD) Pol Foundation Yerala Medical Trust (YMT) Dental College and Hospital, Navi Mumbai, IND

**Keywords:** artificial intelligence, computer-assisted image interpretation, deep learning, dental imaging, dental radiography, diagnostic imaging, machine learning, oral radiology, radiographic image interpretation, systematic review

## Abstract

The rapid adoption of digital radiography in dentistry has generated large volumes of imaging data, creating opportunities for artificial intelligence (AI)-based tools to assist in diagnostic interpretation. AI, particularly machine learning and deep learning models, has shown promising applications in improving diagnostic efficiency, consistency, and image analysis in dental imaging. This systematic review aimed to evaluate the applications, diagnostic performance, and limitations of AI in dental imaging interpretation.

A systematic literature search was conducted in accordance with Preferred Reporting Items for Systematic Reviews and Meta-Analyses (PRISMA) guidelines using PubMed (MEDLINE) and ScienceDirect databases for studies published between May 2005 and March 2024, employing relevant MeSH terms related to artificial intelligence and dental imaging. Following database-specific search refinement and application of predefined eligibility criteria, 25 studies were screened, of which seven met the final eligibility criteria and were included in the qualitative synthesis.

The findings indicate that AI applications in dental imaging, including caries detection, periodontal bone loss assessment, impacted third molar evaluation, root fracture detection, working length determination, and image registration, demonstrated supportive diagnostic performance and improved efficiency compared with conventional interpretation methods. Although AI systems are not a replacement for clinical expertise, they may serve as useful decision-support tools in dental radiology.

Overall, within the limited evidence base included in this review, AI-assisted tools showed potential to support diagnostic workflows and image interpretation in dental radiology. However, because only a small number of heterogeneous studies were included, these findings should be interpreted cautiously, and further high-quality research and external validation studies are required before widespread clinical implementation.

## Introduction and background

Rapid technological advancements have transformed dentistry, particularly in diagnostic radiology. Digital dental radiography is now integral to clinical practice, offering improved image quality, reduced radiation exposure, and enhanced diagnostic support. In recent years, artificial intelligence (AI) has gained increasing attention in healthcare for its ability to analyze complex datasets and assist clinical decision-making [1].

In dentistry, computer-aided diagnosis (CAD) systems based on machine learning and deep learning, particularly convolutional neural networks (CNNs), have demonstrated applications in radiographic interpretation [2,3]. Classical machine learning methods generally rely on manually extracted image features, whereas deep learning systems automatically learn relevant patterns directly from imaging data. Reported applications include detection and classification of dental caries, tooth identification and numbering, assessment of impacted and erupting third molars, detection of apical lesions, periodontal bone loss segmentation, and maxillary sinus evaluation [2-6]. These applications highlight the potential of AI-assisted systems to support diagnostic efficiency and consistency in oral radiology.

Despite these advances, concerns remain regarding the “black box” nature of AI-based decision-making and the interpretability of complex algorithms in clinical imaging applications [4]. Regulatory frameworks, including the General Data Protection Regulation, further emphasize the importance of transparency in AI-assisted clinical decision-making [7]. Recent analyses indicate that a significant proportion of AI research in dentistry is focused on radiology, accounting for approximately 26.4% of applications [8,9].

The transition from film-based to digital radiography has enabled advanced image processing and AI integration, although software-based processing may introduce diagnostic artifacts [1]. While several AI-driven tools and algorithms, including artificial neural networks and image registration techniques, have demonstrated promising performance, their broader clinical implementation remains limited by challenges related to dataset diversity, external validation, methodological heterogeneity, and ethical considerations [6,10-14].

This systematic review aims to evaluate the applications, diagnostic utility, and limitations of AI-assisted interpretation in digital dental radiography by analyzing relevant literature published between 2005 and 2024.

## Review

Methods

Study Design

This systematic review was conducted following the Preferred Reporting Items for Systematic Reviews and Meta-Analyses (PRISMA) guidelines. The review protocol was prospectively registered with the International Prospective Register of Systematic Reviews (PROSPERO) under the registration number CRD420250626843. The review question was formulated using the Population, Intervention, Comparison, and Outcome (PICO) framework, which is reflected in the study title (Table 1).

**Table 1:** PICO framework for the review question PICO: Population, Intervention, Comparison, and Outcome

Problem/population	Dental patients undergoing radiographic image interpretation
Intervention	AI-assisted interpretation of digital images
Comparison	Interpretation by dental radiologists
Outcomes	Diagnostic accuracy and efficiency of AI-assisted interpretation

Search Strategy

An electronic literature search was conducted using PubMed (MEDLINE) and ScienceDirect databases for studies published between May 2005 and March 2024. A combination of MeSH terms and keywords, including “Artificial Intelligence” AND “Dental Imaging” AND “Image Interpretation, Computer-Assisted,” was used. Boolean operators were applied to refine the search strategy.

Database-specific filters were applied prior to screening to improve relevance, including publication year, language (English), and subject area (medicine and dentistry). Additional filtering based on article type and predefined eligibility criteria was performed, particularly in ScienceDirect, to obtain studies aligned with the review objective. These refinement steps were applied before formal title and abstract screening to improve alignment of retrieved studies with the predefined review objective. The detailed ScienceDirect search strategy is provided in Appendix A, and the PubMed search strategy is provided in Appendix B. The search was limited to PubMed and ScienceDirect. Therefore, relevant studies indexed exclusively in other databases such as Scopus, Web of Science, Embase, or the Cochrane Library may not have been captured, and this is acknowledged as a limitation of the review.

Eligibility Criteria

Inclusion criteria: Studies were included if they evaluated the application of artificial intelligence in dental imaging interpretation, involved dental radiographic modalities, and were published as full-text articles in English. Original research studies and selected literature directly relevant to AI-assisted dental image interpretation were considered for inclusion.

Exclusion criteria: Studies not related to dental imaging or artificial intelligence-assisted image interpretation were excluded. Animal and cadaver studies, studies involving non-dental imaging modalities or unrelated specialties, bibliometric studies, case reports, and studies focusing primarily on educational, learning-based, or non-clinical applications were excluded. General review articles not directly relevant to AI-assisted dental image interpretation, as well as studies lacking sufficient or relevant data on AI-assisted dental image interpretation, were also excluded.

Study Selection

Records were screened based on titles and abstracts after confirmation that no duplicate studies were identified across the selected databases and after application of predefined inclusion criteria. Studies were excluded at this stage if they were irrelevant to the study objective or did not meet the eligibility criteria. Full-text articles were retrieved for all potentially eligible studies and further assessed for inclusion. Two reviewers independently performed the screening and selection process, and any disagreements were resolved through discussion.

Data Extraction

Data were extracted independently by two reviewers using a standardized data extraction form. Extracted variables included author details, year of publication, study population, imaging modality, type of AI model used, study objectives, and key outcomes. Any discrepancies between reviewers were resolved through discussion. Due to substantial heterogeneity in AI models, imaging modalities, datasets, study objectives, comparison groups, and reported outcome measures across the included studies, quantitative synthesis or meta-analysis was considered inappropriate. Therefore, findings were synthesized narratively, with emphasis on diagnostic performance, efficiency, and methodological characteristics reported by individual studies.

Risk of Bias Assessment Method

Risk of bias was assessed using the Quality Assessment of Diagnostic Accuracy Studies-2 (QUADAS-2) tool for studies evaluating diagnostic accuracy [15]. The domains assessed included patient selection, index test, reference standard, and flow and timing. QUADAS-2 was not applied to methodological or non-diagnostic studies because the tool was not appropriate for those study designs. These studies were summarized narratively with attention to study design, methodological clarity, dataset characteristics, and relevance to the review objective.

Results

Following database-specific search refinement and application of predefined eligibility criteria, 25 records from PubMed and ScienceDirect were screened based on titles and abstracts. No duplicates were identified between the two databases. Following title and abstract screening, 17 studies were excluded. Eight full-text articles were assessed for eligibility, of which one study was excluded for not meeting the final eligibility criteria.

In PubMed, a Boolean AND combination of three MeSH terms (“Artificial Intelligence,” “Dental Imaging,” and “Image Interpretation, Computer-Assisted”) refined the results to eight studies. In ScienceDirect, the initial 1,700 records were reduced to 17 studies through sequential filtering based on publication years (2005-2024), English language, subject area (medicine and dentistry), article type, and predefined eligibility criteria.

**Figure 1:**
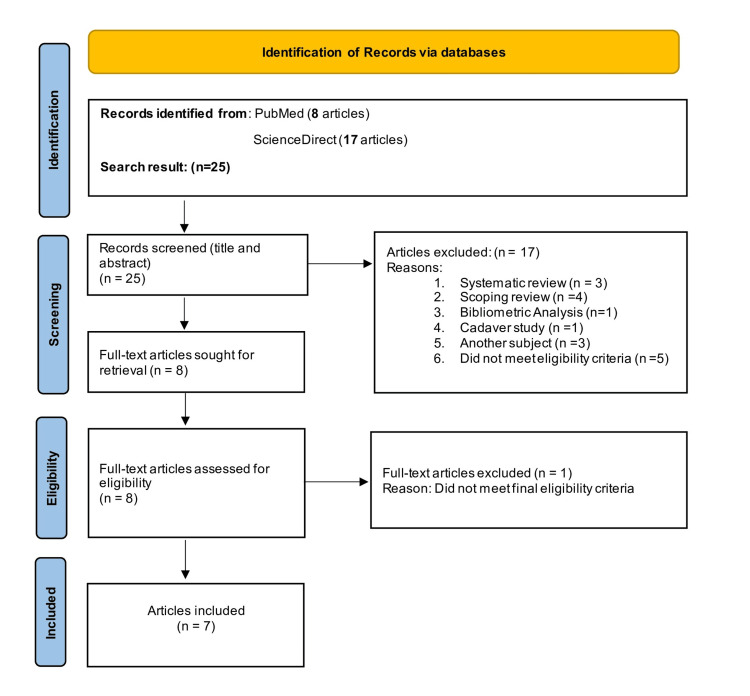
PRISMA flowchart showing article selection for evaluation of the use of artificial intelligence in dental imaging interpretation PRISMA: Preferred Reporting Items for Systematic Reviews and Meta-Analyses

Ultimately, seven studies met the final eligibility criteria and were included in the qualitative synthesis, comprising six studies from PubMed and one from ScienceDirect (Figure 1). Therefore, the findings of this review should be interpreted as a focused qualitative synthesis of a limited number of eligible studies rather than a comprehensive representation of all AI applications in dental imaging.

The included studies demonstrated applications of AI in dental imaging, including automated segmentation, caries detection, periodontal bone loss assessment, root fracture detection, working length determination, image registration, and impacted third molar evaluation. Most studies showed improved diagnostic performance and efficiency with AI-assisted interpretation compared to conventional methods (Table 2).

**Table 2:** Summary of included studies evaluating artificial intelligence in dental imaging interpretation

Study	Authors	Year	Sample size	Population	AI model used	Aim of the article	Comparison	Outcomes
1.	Li te al. [11]	2005	Not clearly reported	Dental X-ray images	Level set methods	To develop automated segmentation methods for identifying bone loss and root decay in dental X-rays.	Manual segmentation methods	Automated segmentation for dental X-rays to identify bone loss and root decay.
2.	Economopoulos et al. [12]	2008	Not clearly reported	Dental image datasets	Enhanced Hexagonal Centre-Based Inner Search (EHCBIS) algorithm	To improve registration accuracy for dental image alignment through a novel algorithm.	Existing image alignment methods	Improved registration accuracy for dental image alignment.
3.	Araki K et al. [6]	2010	50 observers	Dental professionals detecting approximal caries	Computer-assisted diagnostic tools	To evaluate the impact of computer-assisted tools on observer performance for approximal caries detection.	Diagnostic performance without assistance	Enhanced observer performance for approximal caries detection in intraoral radiographs.
4.	Saghiri et al. [13]	2012	60 radiographs	Radiographs of minor apical foramina	Artificial Neural Networks (ANNs)	To determine the accuracy of ANNs in locating the minor apical foramen in radiographs.	Manual localization by experts	Accurate localization of the minor apical foramen with 93% accuracy.
5.	Kositbowornchai et al. [14]	2013	120 extracted teeth	Teeth with suspected vertical root fractures	Artificial Neural Networks (ANNs)	To assess the effectiveness of ANNs in detecting vertical root fractures in digital radiographs.	Manual interpretation by radiologists	High sensitivity (98%) and accuracy (95.7%) for detecting vertical root fractures.
6.	Yoon et al. [1]	2018	Not clearly reported	Radiographic datasets	Digital radiographic processing algorithms	To explore the benefits and challenges of digital radiographic image enhancement.	Non-enhanced radiographic interpretation	Highlighted benefits and challenges of digital image enhancement in dental diagnostics.
7.	Faadiya et al. [3]	2024	Not clearly reported	Patients with impacted third molars	Deep learning models	To evaluate the diagnostic accuracy of deep learning models for impacted third molars using panoramic radiographs.	Traditional radiographic interpretation	Improved diagnostic accuracy for impacted third molars in panoramic radiographs; accuracy up to 99%.

Risk of Bias Findings

The QUADAS-2 assessment findings for the diagnostic accuracy studies are summarized in Table 3. Variable risk of bias was observed across the assessed domains, particularly in reference standard and flow and timing. Publication bias could not be formally assessed; however, selective reporting cannot be excluded. Overall methodological heterogeneity and limited external validation were noted across studies.

**Table 3:** QUADAS-2 risk of bias summary for included diagnostic accuracy studies 🟢 Low risk | 🟡 Unclear risk | 🔴 High risk Quality Assessment of Diagnostic Accuracy Studies‑2 (QUADAS-2) assessment was performed only for studies evaluating the diagnostic accuracy of artificial intelligence in dental imaging.

Study	Patient selection	Index test	Reference standard	Flow and timing	Overall
Araki et al. (2010) [6]	🔴 High	🟢 Low	🟢 Low	🟢 Low	🟡 Moderate
Kositbowornchai et al. (2013) [14]	🟢 Low	🟢 Low	🟢 Low	🔴 High	🟡 Moderate
Saghiri et al. (2012) [13]	🟢 Low	🔴 High	🟡 Unclear	🟢 Low	🔴 High

For studies not evaluating diagnostic accuracy (n = 4), no formal standardized quality appraisal tool was applied, as QUADAS-2 was not suitable for these study designs. These studies were summarized narratively, with commonly noted methodological limitations including lack of external validation, dataset variability, and heterogeneity in study design.

Discussion

This systematic review evaluates the role of AI in dental imaging and highlights its growing applications in diagnostic image interpretation. Across the included studies, AI-based systems supported image interpretation and assisted clinicians in diagnostic workflows. However, because only seven studies were included across the selected search period, broad conclusions regarding the overall effectiveness of AI in dental imaging should be made cautiously.

AI applications in dental radiography primarily rely on deep learning and machine learning algorithms for automated image analysis. Notable uses include caries detection and classification, where AI systems analyze bitewing and periapical radiographs to identify interproximal caries, assess lesion depth, and reduce false-positive diagnoses by accounting for projection geometry and artifacts [6]. AI also demonstrates utility in periodontal disease assessment by measuring alveolar bone levels, detecting vertical and horizontal bone loss, and enabling automated segmentation of bone loss and root decay [1,11].

In oral surgery and endodontics, AI models trained on panoramic radiographs show strong performance in assessing impacted mandibular third molars and their relationship with the inferior alveolar nerve, supporting treatment planning [3]. Artificial neural networks have also been applied in locating the apical foramen and detecting vertical root fractures compared with conventional interpretation [13,14,16]. Image registration algorithms further support longitudinal assessment by accurately aligning sequential radiographs [12].

Broader literature has reported potential AI applications in cone-beam computed tomography (CBCT) imaging, including automated nerve tracing, artifact reduction, and three-dimensional anatomical mapping; however, CBCT-specific evidence within the included studies was limited. AI systems function as decision-support tools that assist diagnostic interpretation, while professional judgment remains essential in patient management and final diagnosis [2,3,5,17].

Quantitative meta-analysis was not performed because the included studies differed substantially in AI models, imaging modalities, target conditions, datasets, comparison methods, and reported outcome measures. As a result, statistical pooling of diagnostic accuracy measures was not considered appropriate, and findings were synthesized narratively (Table 3). Another limitation is that only PubMed and ScienceDirect were searched; therefore, the review may not include all relevant studies available in other major databases. Variability in training datasets, imaging protocols, and limited external validation may affect generalizability. Publication bias cannot be excluded, particularly in technology-driven studies.

Future research should focus on standardizing methodologies, improving dataset quality and diversity, and ensuring transparent reporting. Rigorous external validation through large-scale, multicenter studies is essential to improve generalizability. Challenges such as limited dataset diversity, lack of standardized validation protocols, ethical concerns related to data privacy, and the interpretability of “black-box” models must be addressed. The development of task-specific and interpretable AI systems, along with their integration into diagnostic workflows, remains important for safe and effective implementation in dental imaging.

## Conclusions

Artificial intelligence is increasingly being explored in dental imaging interpretation, with reported applications in diagnostic efficiency, consistency, and image analysis. Within the limited number of studies included in this review, AI-assisted systems showed potential as supportive tools in oral medicine and radiology, particularly for diagnostic workflows and image interpretation. However, the small number of included studies, methodological heterogeneity, limited external validation, and restriction of the search to PubMed and ScienceDirect limit the strength of conclusions that can be drawn.

Further high-quality studies with standardized methodologies, diverse datasets, broader database coverage, and external validation are required before AI-assisted systems can be widely recommended for routine clinical implementation in dental radiology.

## Appendices

Appendix A 

**Table 4:** ScienceDirect search strategy

Step	Search/filter applied	Description	Records remaining
1	Initial search	Keywords: “artificial intelligence” AND “dental imaging” AND “image interpretation”	1,700
2	Date filter	Publication years limited to 2005–2024	1,331
3	Language filter	English language only	1,328
4	Subject area filter	Medicine and Dentistry	766
5	Article type screening	ScienceDirect article type filter: Review articles	189
6	Eligibility screening	Studies not meeting predefined inclusion criteria related to AI-assisted dental imaging interpretation were excluded.	29
7	Full-text availability	Open-access articles assessed for eligibility	17
8	Final inclusion	Studies meeting all inclusion criteria	1

Appendix B 

**Table 5:** PubMed search strategy

Step	Search component	Search terms/strategy	Records retrieved
1	#1 Artificial intelligence	“Artificial Intelligence”[MeSH]	20819
2	#2 Dental imaging	“Dental Imaging” OR “Radiography, Dental”[MeSH]	171
3	#3 Image interpretation	“Image Interpretation, Computer-Assisted”[MeSH]	43401
4	Combined search	#1 AND #2 AND #3	8
5	Filters applied	English language, publication years 2005–2024	8
6	Final inclusion	Studies meeting eligibility criteria	6
